# Association of Coffee Consumption with Hearing and Tinnitus Based on a National Population-Based Survey

**DOI:** 10.3390/nu10101429

**Published:** 2018-10-04

**Authors:** Sang-Youp Lee, Gucheol Jung, Myoung-jin Jang, Myung-Whan Suh, Jun Ho Lee, Seung Ha Oh, Moo Kyun Park

**Affiliations:** 1 Department of Otorhinolaryngology-Head and Neck Surgery, Seoul National University College of Medicine, Seoul 03080, Korea; lsy738@hanmail.net (S.-Y.L.); drmung@naver.com (M.-W.S.); junlee@snu.ac.kr (J.H.L.); shaoh@snu.ac.kr (S.H.O.); 2 Medical Research Collaborating Center, Seoul National University Hospital, Seoul 03080, Korea; 9E887@snuh.org (G.J.); mjjang2014@naver.com (M.-j.J.); 3 Sensory Organ Research Institute, Seoul National University Medical Research Center Seoul, Seoul 03080, Korea

**Keywords:** adult, coffee, hearing, protection, tinnitus

## Abstract

Coffee is the one of the most common beverages worldwide and has received considerable attention for its beneficial health effects. However, the association of coffee with hearing and tinnitus has not been well studied. The aim of this study was to investigate the association of coffee with hearing and tinnitus based on a national population-based survey. We evaluated hearing and tinnitus data from the 2009–2012 Korean National Health and Nutrition Examination Survey and their relationship with a coffee consumption survey. All patients underwent a medical interview, physical examination, hearing test, tinnitus questionnaire and nutrition examination. Multivariable logistic regression models were used to examine the associations between coffee and hearing loss or tinnitus. We evaluated 13,448 participants (≥19 years) participants. The frequency of coffee consumption had a statistically significant inverse correlation with bilateral hearing loss in the 40–64 years age group. Daily coffee consumers had 50–70% less hearing loss than rare coffee consumers, which tended to be a dose-dependent relationship. In addition, the frequency of coffee consumption had an inverse correlation with tinnitus in the 19–64 years age group but its association was related with hearing. Brewed coffee had more of an association than instant or canned coffee in the 40–64 years age group. These results suggest a protective effect of coffee on hearing loss and tinnitus.

## 1. Introduction

Coffee is the most commonly consumed beverage, apart from water, in the world [1]. Coffee and its compounds have various effects on human health [1,2]. Coffee consumption has been associated with a decreased risk of cancers [3], diabetes [4], Parkinson’s disease [5], liver disease [6] and cardiovascular disease [7]. It has also been associated with low birth weight, preterm birth [8] and fractures in women [9,10]. Dementia and depression are related to coffee consumption [11,12]. However, its effect could be related to coffee consumption behavior, social relationships and culture. The health effects of coffee have not received much consideration. Actually, about half of consumers believe drinking coffee is bad for their health [13].

Coffee contains over 1000 bioactive compounds [14,15] with functions, including antioxidant, anti-inflammatory, anti-fibrotic and anticancer effects [1]. In addition, coffee contains polyphenols, such as caffeic acid and caffeic acid phenethyl ester, which have antioxidant effects and protect against hearing loss in vivo and in vitro [16,17]. Caffeine is an important component of coffee that varies according to the preparation method [1].

Hearing loss is a major public health problem [18,19]. One-fifth of adults suffer from hearing loss if mild and unilateral hearing loss are included [20]. Hearing loss is third in terms of disease burden [21]. Common causes of hearing loss are age and noise exposure. Hearing loss affects communication and relationships with people. In particular, it affects talking and has been associated with depression and anxiety [22]. Hearing loss is associated with decreased cognitive performance and dementia [23].

Tinnitus is not a single disease entity. Actually, it is a symptom that decreases quality of life and is related with hearing loss and aging. The prevalence of tinnitus is 12–30% worldwide [24,25]. In addition, coffee and caffeine are often blamed as a cause of tinnitus [26,27]. However, the effect of caffeine on tinnitus remains controversial [28,29]. Few large population-based studies have investigated the effect of coffee consumption on hearing and tinnitus [30].

The aim of this study was to investigate the association of coffee with hearing and tinnitus in adult and elderly participants based on a national population-based survey. We compared the consumption frequency and type of coffee and the prevalence of hearing loss and tinnitus.

## 2. Materials and Methods

### 2.1. Study Population

This study used data from the Korean National Health and Nutrition Examination Survey (KNHANES). This survey collects information, such as health and nutritional status, from a representative sample of the general Korean population to assess the health-related behavior, health condition and nutritional state of Koreans.

The subjects were asked about their hearing, symptoms of tinnitus, health behavior and nutrition by questionnaire. The participants were asked about the annoyance of tinnitus measured by the following answers: “No,” “slightly annoying” and “very annoying and difficult to sleep.” Information about the subjects included sleep time, stress, education level (less than middle school or beyond high school), education level of the parents, income (<25%, 25–50%, 50–75%, or >75% according to the equivalized household income per month), current smoking status and alcohol drinking status (social drinker, heavy drinker, or problem drinker). Health status (hypertension, diabetes, anemia, renal failure, thyroid disease, osteoporosis and menopause) was also checked. Duration of occupational exposure to noise and earphone and headphone use time were measured.

Physical examinations were conducted by a physician to assess any problems with the tympanic membrane or other ear, nose and throat problems, including perforation or retraction of the tympanic membrane, otitis media with effusion and cholesteatoma. Pure tone audiometry was performed at 0.5, 1, 2, 3, 4 and 6 kHz in a soundproof room. The severity of hearing loss was based on a lower threshold of unilateral hearing loss and a higher threshold for bilateral hearing loss. The pure tone average was the average of the hearing levels at 0.5, 1, 2 and 3 kHz or 0.5, 1, 2 and 4 kHz, whereas the high frequency hearing level was the average of the hearing levels at 3, 4 and 6 kHz. Blood samples were collected and analyzed in a single laboratory (Neodin Medical Institute, Seoul, Korea).

**Figure 1:**
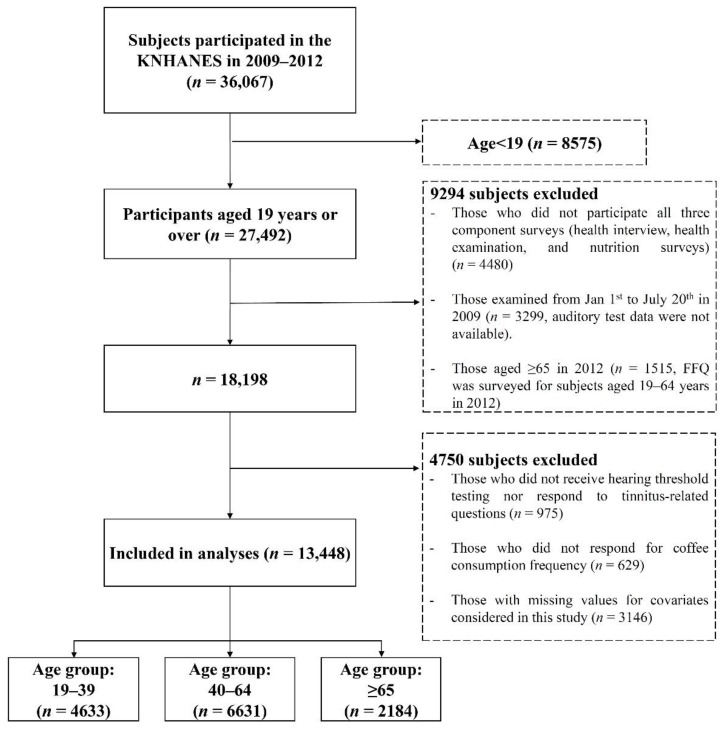
Flow chart of the selection process. KNHANES, Korean National Health and Nutrition Examination Survey.

In total, 36,067 individuals participated in the 2009–2012 KNHANES. Individuals with ear disease (external ear problem, middle ear problem, inner ear problem, retrocochlear problem, congenital hearing loss and systemic disease) were not included here. Of them, 27,492 participants were age ≥19 years. Among 27,492 participants aged ≥19, 9294 participants were excluded because they did not complete all three component surveys (health interview, health examination, and nutrition surveys) (n = 4480) or examined from January 1 to July 20 in 2009 (n = 3299, auditory test data were not available) or aged ≥ 65 in 2012 (n = 1515, FFQ was surveyed for subjects aged 19–64 years in 2012). Of the remaining 18,198 subjects, additional 4750 were excluded because they did not receive hearing threshold testing nor respond to tinnitus-related questions (n = 975) or did not respond for coffee consumption frequency (n = 629) or have missing values for covariates considered in this study (n = 3146). Finally, 13,448 subjects (4633 subjects aged 19–39, 6631 aged 19–39 and 2184 aged ≥65 years) were included in the analysis for the present study (Figure 1). This study was approved by the Institutional Review Board of the Seoul National University Hospital (IRB number: E-1808-064-965).

### 2.2. Assessment of Coffee Consumption

Coffee consumption frequency was assessed using the food-frequency questionnaire (FFQ). Participants were asked to indicate how frequently they consumed coffee over the previous year based on ten categories (none, 6–11 times per year, once per month, two to three times per month, once per week, two to three times per week, four to six times per week, once per day, twice per day and three times per day) in 2009–2011 and on nine categories (never or seldom, once per month, two to three times per month, once per week, two to four times per week, five to six times per week, once per day, twice per day and three times per day) in 2012, in which the first two categories in the previous FFQ version were combined into “never or seldom” and four times per week was grouped into two or three times per week.

Coffee consumption frequency was categorized into rarely, monthly, weekly and daily using the FFQ data as follows: rarely, less than once per month; monthly, one to three times per month; weekly, one to six times per week; and daily, once or more per day.

The information on the types and amount of all coffee that participants consumed over the past 24 h was collected by trained dietitians 1 week after the health interview. The type of coffee was grouped into brewed, instant, or canned coffee using the 24-hour dietary recall method.

### 2.3. Statistical Analysis

The subjects’ characteristics according to coffee consumption frequency are presented as median (interquartile range) or number (proportion) and compared using the Fisher exact test (binary covariates), the chi-square test (more than three categories), or the Wilcoxon rank-sum test (continuous covariates). Multivariable logistic regression models were used to examine the associations between coffee consumption and hearing loss or tinnitus. The analyses were adjusted for the following potential confounders: age, sex, education, parents’ education, perceived stress, exposure to indoor secondhand smoke, current smoking, heavy drinking, drinking-related problems, menopause, history of hypertension, diabetes mellitus, anemia, kidney failure, thyroid disorder, tympanic membrane perforation, cholesteatoma and otitis media with effusion. The multivariable models for tinnitus or annoyance related to tinnitus included hearing loss as well as the potential covariates described above. To examine the association of the type of coffee consumed with hearing loss and tinnitus, multivariable logistic regression analyses were performed for the adjusted associations between coffee type consumed and hearing loss or tinnitus. All statistical analyses were performed using SAS software (version 9.2; SAS Institute, Cary, NC, USA).

## 3. Results

The prevalence rates of unilateral and bilateral hearing loss in the study population were 1.19% and 0.17% for subjects in the 19–39 years age group, 5.01% and 2.9% for subjects in the 40–64 years age group and 14.24% and 20.97% for subjects in the ≥65 years age group. The prevalence rates of tinnitus and tinnitus-related annoyance were 18.07% and 3.86% for subjects in the 19–39 years age group, 19.92% and 6.24% for subjects in the 40–64 years age group and 27.98% and 12.82% for subjects in the ≥65 years age group (Table 1). The participants’ characteristics according to age group and the frequency of coffee consumption showed that there were differences in the covariates according to coffee consumption: age, sex, educational level, house income, sleeping duration, stress, exposure to indoor secondhand smoke, current smoking, heavy drinking, difficulties controlling alcohol use, menopause, hypertension, diabetes, kidney failure and thyroid disorder (Table S1).

Table 2 shows the results of the association between coffee consumption and hearing loss. No significant correlation was detected between coffee consumption frequency and unilateral hearing loss across all age groups. No significant correlation was detected between bilateral hearing loss and coffee consumption frequency in the 19–39 and ≥65 years age groups. However, daily coffee consumption resulted in a significantly decreased risk of bilateral hearing loss in the 40–64 years age group, compared with the rare consumption group (adjusted odds ratio (aOR), 0.50; 95% confidence interval (CI), 0.33–0.78; *p* = 0.0021), whereas monthly or weekly consumers did not show a significant difference relative to rare consumers. In the 40–64 years age group, odds ratio of mild and moderate hearing loss in daily coffee consumers and mild hearing loss in weekly coffee consumers were significantly lower than those of rare coffee consumers (Table S7). In addition, as the frequency of coffee consumption increased there tended to be a decrease in bilateral hearing loss in the 40–64 years age group.

Table 3 shows the results of the association between coffee consumption and tinnitus and tinnitus-related annoyance. In the univariable analysis, the prevalence of tinnitus in daily coffee consumers was lower than that in the rare coffee consumers in the 19–39 years (unadjusted OR, 0.77; 95% CI, 0.62–0.96; *p* = 0.0186) and 40–64 years (unadjusted odds ratio (OR), 0.81; 95% CI, 0.67–0.99; *p* = 0.0357) age groups. However, in the multivariable models adjusted for potential confounders, the relationships between daily coffee consumers and rare consumers were not significant in the 19–39 year (aOR, 0.80; 95% CI, 0.63–1.00; *p* = 0.0548) and the 40–64 years age groups (aOR, 0.90; 95% CI, 0.73–1.10; *p* = 0.3066). An inverse association was observed between tinnitus-related annoyance and coffee consumption in weekly coffee consumers aged 19–39 years (unadjusted OR, 0.56; 95% CI, 0.33-0.95; *p* = 0.0298) and daily coffee consumers aged ≥ 65 years (unadjusted OR, 0.63; 95% CI, 0.46–0.85; *p* = 0.0026). However, the associations in weekly coffee consumers aged 19–39 (aOR, 0.58; 95% CI, 0.34–1.01; *p* = 0.0529) and daily coffee consumers aged ≥ 65 years (aOR, 0.77; 95% CI, 0.54–1.09; *p* = 0.1355) were not significant in the multivariable analysis.

We investigated associations between types of coffee and hearing loss, tinnitus and tinnitus-related annoyance using multivariable analysis. Table 4 shows “adjusted” odds ratio of hearing loss, tinnitus and tinnitus-related annoyance for three types of coffee. The odds of unilateral hearing loss or tinnitus-related annoyance did not reach statistical significance for all age groups. However, the odds ratio of bilateral hearing loss for brewed coffee in 40–64 years age group is significantly lower than 1 (aOR, 0.61; 95% CI, 0.44–0.84; *p* = 0.0028). And the odds ratio of tinnitus for brewed coffee in 19–39 years age group is significantly lower than 1 (aOR, 0.82; 95% CI, 0.70–0.97; *p* = 0.0175). Contrary, the odds ratio of tinnitus for canned coffee in 40–64 years age group is significantly higher than 1 (aOR, 1.49; 95% CI, 1.17–1.90; *p* = 0.0011).

## 4. Discussion

This study demonstrated the inverse correlation of the frequency of coffee consumption with hearing loss in middle aged Koreans. The prevalence of bilateral hearing loss in daily coffee consumers was significantly lower in the 40–64 years age group compared to the other age groups. However, no significant correlation was observed between unilateral hearing loss and coffee consumption in any other age group. Tinnitus and tinnitus-related annoyance were not related with coffee consumption. Instant coffee consumers aged 40–64 years had less hearing loss and less tinnitus-related annoyance than those in the ≥65 years age group and for any type of coffee.

No previous large-scale study has demonstrated the effects of coffee on hearing loss and tinnitus. Many people believe that coffee has a harmful effect on hearing. Actually, there is a report that caffeine in coffee has detrimental effects on recovery from acoustic overstimulation events [31,32]. Caffeine, a major ingredient of coffee, is proved to be an aggravating factor of Meniere’s disease [33]. Many otologists prescribe a coffee restriction for cases of hearing loss from Meniere’s disease [34]. Some studies have indicated that coffee has preventive effects on hearing loss but most studies were conducted on a specific type of hearing loss or situation. Chang et al. reported that noise-induced hearing loss in workers is less frequent in tea or coffee consumers (OR = 0.03, 95% CI: 0.01–0.51) [35]. Caffeine improves transmission in the peripheral and central brain auditory pathways [36]. Caffeine improves auditory processing in preterm infants, resulting in improved neurodevelopmental outcomes [37]. Hong et al. reported that coffee ameliorates the hearing threshold shift and delayed latency of auditory evoked potentials in patients with diabetic neuropathy [38]. Coffee improves auditory neuropathy in diabetic mice. In addition, trigonelline—the main active compound in coffee extracts—facilitates recovery from pyridoxine-induced auditory neuropathy in a mouse model [39].

Our univariable analyses determined that the prevalence of tinnitus in daily coffee consumers in the 19–39 and 40–64 years age groups was lower than that in rare coffee consumers of the same age groups. The prevalence of tinnitus in weekly coffee consumers age ≥65 years was lower than that in rare coffee consumers in the same age group. However, this tendency disappeared in the multivariable analysis, suggesting that coffee consumption itself does not have a direct correlation with tinnitus. Some covariates, such as bilateral hearing loss, stress and sleep, can indirectly affect tinnitus.

The relationship between coffee and tinnitus is controversial. There is an opinion that caffeine in coffee stimulates ascending auditory pathways or reduces the suppressive effect on the central nervous system, which evokes tinnitus [40]. Other studies have argued that the stimulation increases the detection of tinnitus through increased arousal or anxiety [41]. Caffeine in coffee is known to have deleterious effects on sleep [42,43] and it can aggravate tinnitus-associated distress [44]. However, one study reported that stopping caffeine intake does not improve tinnitus symptoms [29]. Another study reported that higher caffeine intake is associated with a lower risk of tinnitus in women [45]. McComack et al. reported that persistent tinnitus decreases with caffeinated coffee consumption (OR = 0.99 per cup/day) and consumption of caffeinated coffee appears to be associated with lower levels of reported transient tinnitus [30].

The relationship between the frequency of coffee consumption and the occurrence of tinnitus in the 19–39 and ≥65 years age groups appeared to be quite different from that of the 40–64 years age group. The abatement of tinnitus in the 40–64 years age group can be explained by a decrease in bilateral hearing loss. However, no significant decreases in bilateral hearing loss were observed in the 19–39 years and ≥65 years age groups. One of the covariates, such as perceived stress, can reduce tinnitus. In fact, an inverse correlation has been reported between perceived stress and tinnitus [46,47,48]. Coffee consumption is associated with social activity; thus, it is highly probable that socially active people have a relatively lower level of perceived stress and low stress can lower the incidence of tinnitus.

Types of coffee have association with hearing loss and tinnitus. Our results suggest that brewed coffee can have preventive effects on bilateral hearing loss and tinnitus but canned coffee can have inducing effect on tinnitus for some age groups. Difference in preparation method, heat treatment (freeze-drying or high temperature sterilization) and expiration date seem to have affected on bioactive constituents in each type of coffee [49,50]. However, since the details of coffee type are very diverse, it is difficult to make uniform conclusion.

We have some limitations in this study. First, this is an observational study, so it is difficult to generalize the result of this study to the causal relationship from coffee consumption to hearing loss and tinnitus. To confirm causality, well-controlled experimental design will be needed. Second, we could not analyze unilateral and bilateral tinnitus separately, because KNHANES data did not discern tinnitus side. However, bilateral tinnitus is different from unilateral one in the point of heritability and prognosis [51]. Therefore, it is desirable to discern the side of tinnitus. Third, the frequency of coffee consumption was estimated by questionnaires of subjects. It depends on the memory of the subjects, so it is possible that there is a measurement error.

## 5. Conclusions

According to the results of KNHANES analyses, coffee consumers had a low prevalence of bilateral hearing loss. However, the path of lower bilateral hearing loss and tinnitus varied according to age group. The incidence of bilateral hearing loss was low in coffee consumers aged 40–64 years, which influenced the low prevalence of tinnitus. However, other covariates, such as the low perceived stress of coffee consumers, seemed to be the main cause for the low prevalence of tinnitus in the 19–39 and ≥65 years age groups. In addition, brewed coffee consumers had lower rate of bilateral hearing loss and tinnitus.

## Figures and Tables

**Table 1:** Characteristics of study subjects in Korean National Health and Nutrition Examination Survey KNHANES (2009–2012) by frequency of coffee consumption.

Symptoms by Group	Frequency of Coffee Consumption				
	Total	Rarely	Monthly	Weekly	Daily
Age group (19–39)	*n* = 4633	*n* = 634	*n* = 304	*n* = 873	*n* = 2822
Hearing loss *, *n* (%)					
Unilateral	55 (1.19%)	11 (1.74%)	2 (0.66%)	6 (0.69%)	36 (1.28%)
Bilateral	8 (0.17%)	1 (0.16%)	0 (0.00%)	3 (0.34%)	4 (0.14%)
Tinnitus, *n* (%)	837 (18.07%)	130 (20.50%)	62 (20.39%)	177 (20.27%)	468 (16.58%)
Tinnitus-related annoyance, *n* (%)	179 (3.86%)	33 (5.21%)	13 (4.28%)	26 (2.98%)	107 (3.79%)
Age group (40–64)	*n* = 6631	*n* = 656	*n* = 308	*n* = 899	*n* = 4768
Hearing loss *, *n* (%)					
Unilateral	332 (5.01%)	40 (6.1%)	15 (4.87%)	47 (5.23%)	230 (4.82%)
Bilateral	192 (2.90%)	31 (4.73%)	18 (5.84%)	33 (3.67%)	110 (2.31%)
Tinnitus, *n* (%)	1321 (19.92%)	149 (22.71%)	64 (20.78%)	191 (21.25%)	917 (19.23%)
Tinnitus-related annoyance, *n* (%)	414 (6.24%)	50 (7.62%)	18 (5.84%)	66 (7.34%)	280 (5.87%)
Age group (≥65)	*n* = 2184	*n* = 429	*n* = 122	*n* = 383	*n* = 1250
Hearing loss *, *n* (%)					
Unilateral	311 (14.24%)	71 (16.55%)	18 (14.75%)	58 (15.14%)	164 (13.12%)
Bilateral	458 (20.97%)	99 (23.08%)	20 (16.39%)	79 (20.63%)	260 (20.80%)
Tinnitus, *n* (%)	611 (27.98%)	135 (31.47%)	38 (31.15%)	95 (24.80%)	343 (27.44%)
Tinnitus-related annoyance, *n* (%)	280 (12.82%)	73 (17.02%)	18 (14.75%)	47 (12.27%)	142 (11.36%)

* Hearing loss ≥ 41 dB for four-frequency average of pure-tone thresholds at 500, 1000, 2000 and 4000 Hz.

**Table 2:** Odds ratios and 95% confidence intervals of coffee consumption for hearing loss.

	Univariable Analysis				Multivariable Analysis *			
Frequency of Coffee Consumption	Hearing Loss (Unilateral)		Hearing Loss (Bilateral)		Hearing Loss (Unilateral)		Hearing Loss (Bilateral)	
	OR (95% CI)	*p*-Value	OR (95% CI)	*p*-Value	OR (95% CI)	*p*-Value	OR (95% CI)	*p*-Value
**Age 19–39**								
Rarely	Reference		Reference		Reference		Reference	
Monthly	0.38 (0.08, 1.70)	0.2039	0.69 (0.03, 17.15)	0.8231	0.42 (0.09, 1.99)	0.2768	0.59 (0.06, 6.35)	0.6656
Weekly	0.39 (0.14, 1.07)	0.0664	1.70 (0.25, 11.55)	0.5880	0.45 (0.16, 1.28)	0.1338	1.53 (0.37, 6.32)	0.5607
Daily	0.73 (0.37, 1.45)	0.3688	0.67 (0.11, 4.29)	0.6765	0.76 (0.36, 1.59)	0.4595	0.74 (0.19, 2.94)	0.6715
**Age 40–64**								
Rarely	Reference		Reference		Reference		Reference	
Monthly	0.79 (0.43, 1.45)	0.4442	1.25 (0.69, 2.28)	0.4604	0.80 (0.42, 1.52)	0.4891	1.19 (0.62, 2.26)	0.6051
Weekly	0.85 (0.55, 1.31)	0.4611	0.77 (0.47, 1.27)	0.3023	0.86 (0.54, 1.36)	0.5141	0.74 (0.43, 1.26)	0.2624
Daily	0.78 (0.55, 1.10)	0.1603	0.48 (0.32, 0.72)	0.0004	0.85 (0.59, 1.23)	0.3865	0.50 (0.33, 0.78)	0.0021
**Age ≥ 65**								
Rarely	Reference		Reference		Reference		Reference	
Monthly	0.87 (0.50, 1.53)	0.6344	0.65 (0.39, 1.11)	0.1155	0.89 (0.48, 1.66)	0.7070	0.72 (0.40, 1.29)	0.2720
Weekly	0.90 (0.62, 1.31)	0.5841	0.87 (0.62, 1.21)	0.3997	0.97 (0.64, 1.47)	0.8765	0.90 (0.62, 1.31)	0.5953
Daily	0.76 (0.56, 1.03)	0.0778	0.88 (0.67, 1.14)	0.3212	0.85 (0.60, 1.19)	0.3360	0.84 (0.62, 1.14)	0.2669

* Adjusted for age, sex, education, parents’ education, perceived stress, exposure to indoor secondhand smoke, current smoking, heavy drinking, drinking-related problem, menopause, history of hypertension, diabetes mellitus, anemia, kidney failure, thyroid disorder, tympanic membrane perforation, cholesteatoma and otitis media with effusion. OR, odds ratio. CI, confidence interval.

**Table 3:** Odds ratios and 95% confidence intervals of coffee consumption for tinnitus.

	Univariable Analysis				Multivariable Analysis *			
Frequency of Coffee Consumption	Tinnitus		Tinnitus-Related Annoyance		Tinnitus		Tinnitus-Related Annoyance	
	OR (95% CI)	*p*-Value	OR (95% CI)	*p*-Value	OR (95% CI)	*p*-Value	OR (95% CI)	*p*-Value
**Age 19–39**								
Rarely	Reference		Reference		Reference		Reference	
Monthly	0.99 (0.71, 1.39)	0.9688	0.81 (0.42, 1.57)	0.5382	1.09 (0.77, 1.54)	0.6366	0.94 (0.48, 1.84)	0.8541
Weekly	0.99 (0.77, 1.27)	0.9129	0.56 (0.33, 0.95)	0.0298	1.05 (0.81, 1.37)	0.7013	0.58 (0.34, 1.01)	0.0529
Daily	0.77 (0.62, 0.96)	0.0186	0.72 (0.48, 1.07)	0.1043	0.80 (0.63 ,1.00)	0.0548	0.76 (0.50, 1.16)	0.2035
**Age 40–64**								
Rarely	Reference		Reference		Reference		Reference	
Monthly	0.89 (0.64, 1.24)	0.4998	0.75 (0.43, 1.31)	0.3159	0.92 (0.65, 1.29)	0.6210	0.75 (0.42, 1.35)	0.3376
Weekly	0.92 (0.72, 1.17)	0.4892	0.96 (0.66, 1.41)	0.8349	0.97 (0.75, 1.25)	0.8249	1.07 (0.72, 1.60)	0.7322
Daily	0.81 (0.67, 0.99)	0.0357	0.76 (0.55, 1.03)	0.0795	0.90 (0.73, 1.10)	0.3066	0.92 (0.66, 1.29)	0.6464
**Age ≥ 65**								
Rarely	Reference		Reference		Reference		Reference	
Monthly	0.99 (0.64, 1.52)	0.9463	0.84 (0.48, 1.48)	0.5530	1.02 (0.63, 1.65)	0.9417	0.94 (0.49, 1.78)	0.8446
Weekly	0.72 (0.53, 0.98)	0.0357	0.68 (0.46, 1.01)	0.0582	0.71 (0.51, 1.00)	0.0514	0.74 (0.48, 1.16)	0.1881
Daily	0.82 (0.65, 1.05)	0.1109	0.63 (0.46, 0.85)	0.0026	0.95 (0.72, 1.24)	0.6899	0.77 (0.54, 1.09)	0.1355

* Adjusted for age, sex, education, parents’ education, perceived stress, exposure to indoor secondhand smoke, current smoking, heavy drinking, drinking-related problem, menopause, history of hypertension, diabetes mellitus, anemia, kidney failure, thyroid disorder, tympanic membrane perforation, cholesteatoma, otitis media with effusion, and hearing loss.

**Table 4:** Odds ratios and 95% confidence intervals of coffee type.

Coffee Type	Hearing Loss (Unilateral)		Hearing Loss (Bilateral)	
	OR * (95% CI)	*p*-Value	OR * (95% CI)	*p*-Value
**Age group: 19–39**				
Brewed coffee (yes vs. no)	0.95 (0.53, 1.69)	0.8599	0.46 (0.16, 1.27)	0.1333
Instant coffee (yes vs. no)	0.65 (0.22, 1.91)	0.4369	0.64 (0.08, 4.88)	0.6683
Canned coffee (yes vs. no)	1.02 (0.39, 2.64)	0.9752	1.28 (0.32, 5.08)	0.7223
**Age group: 40–64**				
Brewed coffee (yes vs. no)	1.04 (0.81, 1.34)	0.7414	0.61 (0.44, 0.84)	0.0028
Instant coffee (yes vs. no)	1.11 (0.81, 1.51)	0.5216	0.70 (0.45, 1.09)	0.1133
Canned coffee (yes vs. no)	1.41 (0.86, 2.30)	0.1694	0.63 (0.23, 1.73)	0.3672
**Age group: ≥65**				
Brewed coffee (yes vs. no)	0.84 (0.64, 1.10)	0.2083	1.02 (0.80, 1.30)	0.8886
Instant coffee (yes vs. no)	0.87 (0.60, 1.26)	0.4472	0.74 (0.53, 1.04)	0.0842
Canned coffee (yes vs. no)	1.38 (0.51, 3.74)	0.5233	0.89 (0.30, 2.64)	0.8294
	**Tinnitus**		**Tinnitus-Related Annoyance**	
	**OR ^†^ (95% CI)**	***p*-Value**	**OR ^†^ (95% CI)**	***p*-Value**
**Age group: 19–39**				
Brewed coffee (yes vs. no)	0.82 (0.70, 0.97)	0.0175	1.09 (0.80, 1.50)	0.5821
Instant coffee (yes vs. no)	1.08 (0.84, 1.41)	0.5439	1.04 (0.62, 1.74)	0.8812
Canned coffee (yes vs. no)	0.95 (0.74, 1.23)	0.7163	1.13 (0.68, 1.86)	0.6454
**Age group: 40–64**				
Brewed coffee (yes vs. no)	1.00 (0.87, 1.14)	0.9359	0.96 (0.77, 1.19)	0.6790
Instant coffee (yes vs. no)	0.96 (0.81, 1.14)	0.6779	0.96 (0.72, 1.27)	0.7660
Canned coffee (yes vs. no)	1.49 (1.17, 1.90)	0.0011	1.28 (0.83, 1.95)	0.2619
**Age group: ≥65**				
Brewed coffee (yes vs. no)	0.92 (0.74, 1.14)	0.4318	0.89 (0.66, 1.20)	0.4441
Instant coffee (yes vs. no)	1.19 (0.90, 1.57)	0.2263	0.95 (0.64, 1.42)	0.7967
Canned coffee (yes vs. no)	0.85 (0.37, 1.97)	0.7051	0.63 (0.14, 2.74)	0.5328

* Adjusted for age, sex, education, parents’ education, perceived stress, exposure to indoor secondhand smoke, current smoking, heavy drinking, drinking-related problem, menopause, history of hypertension, diabetes mellitus, anemia, kidney failure, thyroid disorder, tympanic membrane perforation, cholesteatoma and otitis media with effusion. ^†^ Adjusted for age, sex, education, parents’ education, perceived stress, exposure to indoor secondhand smoke, current smoking, heavy drinking, drinking-related problem, menopause, history of hypertension, diabetes mellitus, anemia, kidney failure, thyroid disorder, tympanic membrane perforation, cholesteatoma, otitis media with effusion and hearing loss.

## Supplementary Materials

The following are available online at http://www.mdpi.com/2072-6643/10/10/1429/s1, Table S1: Characteristics of study subjects in KNHANES (2009–2012) by coffee consumption and age group (19–39), Table S2: Characteristics of study subjects in KNHANES (2009–2012) by coffee consumption and age group (40–64), Table S3: Characteristics of study subjects in KNHANES (2009–2012) by coffee consumption and age group (≥65), Table S4: Degree of unilateral hearing loss by coffee consumption, Table S5: Degree of bilateral hearing loss by coffee consumption, Table S6: Odds ratio and 95% confidence intervals by coffee consumption for degree of unilateral hearing loss, Table S7: Odds ratio and 95% confidence intervals by coffee consumption for degree of bilateral hearing loss.
