# Supplementary material for: Association of Coffee Consumption with Hearing and Tinnitus Based on a National Population-Based Survey
Source: Nutrients. 2018 Oct 4;10(10):1429. doi: 10.3390/nu10101429 (PMC6213338; doi:10.3390/nu10101429)
Supplement: Supplementary file 1 [file nutrients-10-01429-s001.pdf]

Supplementary Tables

Table S1. Characteristics of study subjects in KNHANES (2009–2012) by coffee consumption and age group (19–39).

	Coffee consumption					P-value[1)]
	Total (n=4633)	None (n=634)	Monthly (n=304)	Weekly (n=873)	Daily (N=2822)	
**Age group (19–39)**						
Age (year), median [IQR]	32 [26, 36]	29 [24, 34]	28 [23, 33]	29 [24, 34]	34 [29, 37]	<.0001
Male, n (%)	1739 (37.54%)	223 (35.17%)	96 (31.58%)	334 (38.26%)	1086 (38.48%)	0.0597
Education, n (%)						<.0001
Less than middle school	34 (0.73%)	6 (0.95%)	3 (0.99%)	4 (0.46%)	21 (0.74%)	
High school	1225 (26.44%)	142 (22.40%)	56 (18.42%)	189 (21.65%)	838 (29.70%)	
College or more	3374 (72.83%)	486 (76.66%)	245 (80.59%)	680 (77.89%)	1963 (69.56%)	
Father’s education, n (%)						<.0001
Less than middle school	2002 (43.21%)	237 (37.38%)	102 (33.55%)	319 (36.54%)	1344 (47.63%)	
High school	1592 (34.36%)	234 (36.91%)	130 (42.76%)	325 (37.23%)	903 (32.00%)	
College or more	935 (20.18%)	149 (23.50%)	70 (23.03%)	214 (24.51%)	502 (17.79%)	
No/non-formal education	104 (2.24%)	14 (2.21%)	2 (0.66%)	15 (1.72%)	73 (2.59%)	
Mother’s education, n (%)						<.0001
Less than middle school	2463 (53.16%)	293 (46.21%)	127 (41.78%)	395 (45.25%)	1648 (58.4%)	
High school	1538 (33.2%)	245 (38.64%)	137 (45.07%)	341 (39.06%)	815 (28.88%)	
College or more	421 (9.09%)	73 (11.51%)	34 (11.18%)	109 (12.49%)	205 (7.26%)	
No/non-formal education	211 (4.55%)	23 (3.63%)	6 (1.97%)	28 (3.21%)	154 (5.46%)	
Monthly house income[2)], median [IQR]	333 [250, 500]	333 [208, 485]	333 [208, 500]	340 [247, 500]	348 [250, 500]	0.0072
Sleeping duration (hours), n (%)						<.0001
< 6	307 (6.63%)	46 (7.26%)	18 (5.92%)	58 (6.64%)	185 (6.56%)	
6–7	1127 (24.33%)	118 (18.61%)	63 (20.72%)	213 (24.40%)	733 (25.97%)	
7–8	1465 (31.62%)	156 (24.61%)	87 (28.62%)	268 (30.70%)	954 (33.81%)	

≥8	1734 (37.43%)	314 (49.53%)	136 (44.74%)	334 (38.26%)	950 (33.66%)	
High perceived stress, n (%)	1483 (32.01%)	200 (31.55%)	80 (26.32%)	277 (31.73%)	926 (32.81%)	0.1381
Exposure to indoor secondhand smoke						
At work, n (%)	344 (7.42%)	32 (5.05%)	11 (3.62%)	60 (6.87%)	241 (8.54%)	0.0004
At home, n (%)	87 (1.88%)	10 (1.58%)	7 (2.30%)	20 (2.29%)	50 (1.77%)	0.6202
Current smoking, n (%)	1069 (23.07%)	88 (13.88%)	42 (13.82%)	186 (21.31%)	753 (26.68%)	<.0001
Heavy drinking[3)], n (%)	757 (16.34%)	80 (12.62%)	31 (10.20%)	129 (14.78%)	517 (18.32%)	<.0001
Difficulties controlling alcohol use, n (%)	374 (8.07%)	52 (8.20%)	12 (3.95%)	76 (8.71%)	234 (8.29%)	0.035
Having drinking-related problems, n (%)	340 (7.34%)	46 (7.26%)	13 (4.28%)	73 (8.36%)	208 (7.37%)	0.1203
Menopause (females)						0.1577
No	2888 (99.79%)	411 (100%)	208 (100%)	538 (99.81%)	1731 (99.71%)	
Yes	6 (0.21%)	0 (0%)	0 (0%)	1 (0.19%)	5 (0.29%)	
Hypertension, n (%)	48 (1.04%)	13 (2.05%)	1 (0.33%)	9 (1.03%)	25 (0.89%)	0.0582
Diabetes mellitus, n (%)	25 (0.54%)	6 (0.95%)	2 (0.66%)	3 (0.34%)	14 (0.5%)	0.3788
Anemia, n (%)	391 (8.69%)	53 (8.69%)	29 (9.83%)	62 (7.36%)	247 (8.98%)	0.4375
Kidney failure, n (%)	1 (0.02%)	0 (0.00%)	0 (0.00%)	0 (0.00%)	1 (0.04%)	>.9999
Thyroid disorder, n (%)	50 (1.08%)	7 (1.1%)	3 (0.99%)	11 (1.26%)	29 (1.03%)	0.9271
Tympanic membrane perforation, n (%)	29 (0.63%)	2 (0.32%)	1 (0.33%)	4 (0.46%)	22 (0.78%)	0.5564
Cholesteatoma, n (%)	36 (0.78%)	4 (0.63%)	2 (0.66%)	7 (0.8%)	23 (0.82%)	0.9825
Otitis media with effusion, n (%)	17 (0.37%)	0 (0.00%)	1 (0.33%)	2 (0.23%)	14 (0.5%)	0.2531

1) P-values from Fisher exact tests (binary covariates), chi-square tests (>3 categories) and Wilcoxon rank-sum tests (continuous covariates); 2) Monthly household income (10,000 Korean Won); 3) Heavy drinking: more than 3 drinks per average and more than twice weekly.

Table S2. Characteristics of study subjects in KNHANES (2009–2012) by coffee consumption and age group (40–64).

	Coffee consumption					
	**Total (n=6631)**	**None (n=656)**	**Monthly (n=308)**	**Weekly (n=899)**	**Daily (N=4768)**	**P-value[1)]**
**Age group (40–64)**						
Age (year), median [IQR]	51 [45, 58]	55 [49, 60]	54 [47, 60]	53 [47, 58]	51 [45, 57]	<.0001
Male, n (%)	2749 (41.46%)	199 (30.34%)	113 (36.69%)	334 (37.15%)	2103 (44.11%)	<.0001

Education, n (%)						<.0001
Less than middle school	2145 (32.35%)	285 (43.45%)	123 (39.94%)	333 (37.04%)	1404 (29.45%)	
High school	2484 (37.46%)	220 (33.54%)	105 (34.09%)	306 (34.04%)	1853 (38.86%)	
College or more	2002 (30.19%)	151 (23.02%)	80 (25.97%)	260 (28.92%)	1511 (31.69%)	
Father's education, n (%)						<.0001
Less than middle school	3510 (52.93%)	314 (47.87%)	167 (54.22%)	453 (50.39%)	2576 (54.03%)	
High school	956 (14.42%)	97 (14.79%)	31 (10.06%)	111 (12.35%)	717 (15.04%)	
College or more	529 (7.98%)	50 (7.62%)	16 (5.19%)	72 (8.01%)	391 (8.20%)	
No/non-formal education	1636 (24.67%)	195 (29.73%)	94 (30.52%)	263 (29.25%)	1084 (22.73%)	
Mother's education, n (%)						<.0001
Less than middle school	3813 (57.50%)	335 (51.07%)	175 (56.82%)	487 (54.17%)	2816 (59.06%)	
High school	439 (6.62%)	25 (3.81%)	15 (4.87%)	39 (4.34%)	360 (7.55%)	
College or more	112 (1.69%)	13 (1.98%)	4 (1.30%)	22 (2.45%)	73 (1.53%)	
No/non-formal education	2267 (34.19%)	283 (43.14%)	114 (37.01%)	351 (39.04%)	1519 (31.86%)	
Monthly house income[4)], median [IQR]	333 [200, 500]	300 [150, 500]	300 [167, 495]	325 [183, 500]	350 [208, 535]	<.0001
Sleeping duration (hours), n (%)						0.0422
< 6	840 (12.67%)	109 (16.62%)	45 (14.61%)	126 (14.02%)	560 (11.74%)	
6–7	1888 (28.47%)	172 (26.22%)	85 (27.60%)	256 (28.48%)	1375 (28.84%)	
7–8	2054 (30.98%)	187 (28.51%)	93 (30.19%)	268 (29.81%)	1506 (31.59%)	
≥8	1849 (27.88%)	188 (28.66%)	85 (27.60%)	249 (27.7%)	1327 (27.83%)	
High perceived stress, n (%)	1676 (25.28%)	172 (26.22%)	77 (25.00%)	217 (24.14%)	1210 (25.38%)	0.8117
Exposure to indoor secondhand smoke						
At work, n (%)	481 (7.25%)	30 (4.57%)	8 (2.60%)	55 (6.12%)	388 (8.14%)	<.0001
At home, n (%)	138 (2.08%)	13 (1.98%)	3 (0.97%)	20 (2.22%)	102 (2.14%)	0.5959
Current smoking, n (%)	1232 (18.58%)	58 (8.84%)	19 (6.17%)	135 (15.02%)	1020 (21.39%)	<.0001

Heavy drinking[5]), n (%)	1309 (19.74%)	85 (12.96%)	47 (15.26%)	173 (19.24%)	1004 (21.06%)	<.0001
Difficulties controlling alcohol use, n (%)	610 (9.20%)	49 (7.47%)	28 (9.09%)	93 (10.34%)	440 (9.23%)	0.2844
Having drinking-related problems, n (%)	379 (5.72%)	29 (4.42%)	17 (5.52%)	60 (6.67%)	273 (5.73%)	0.3091
Menopause (females)						<.0001
No	1801 (46.39%)	144 (31.51%)	68 (34.87%)	226 (40.00%)	1363 (51.14%)	
Yes	2081 (53.61%)	313 (68.49%)	127 (65.13%)	339 (60.00%)	1302 (48.86%)	
Hypertension, n (%)	1257 (18.96%)	161 (24.54%)	67 (21.75%)	182 (20.24%)	847 (17.76%)	0.0002
Diabetes mellitus, n (%)	467 (7.04%)	71 (10.82%)	27 (8.77%)	68 (7.56%)	301 (6.31%)	0.0003
Anemia, n (%)	546 (8.46%)	60 (9.66%)	17 (5.74%)	73 (8.45%)	396 (8.47%)	0.2566
Kidney failure, n (%)	17 (0.26%)	4 (0.61%)	1 (0.32%)	6 (0.67%)	6 (0.13%)	0.0043
Thyroid disorder, n (%)	182 (2.74%)	29 (4.42%)	12 (3.90%)	28 (3.11%)	113 (2.37%)	0.0106
Tympanic membrane perforation, n (%)	147 (2.22%)	19 (2.90%)	6 (1.95%)	27 (3.00%)	95 (1.99%)	0.1538
Cholesteatoma, n (%)	141 (2.13%)	19 (2.90%)	5 (1.62%)	17 (1.89%)	100 (2.10%)	0.5032
Otitis media with effusion, n (%)	35 (0.53%)	7 (1.07%)	0 (0.00%)	2 (0.22%)	26 (0.55%)	0.0993

1) P-values from Fisher exact test (binary covariates), chi-square test (>3 categories) and Wilcoxon rank-sum test (continuous covariates); 2) Monthly household income (10,000 Korean Won); 3) Heavy drinking: more than 3 drinks on average and more than twice weekly.

Table S3. Characteristics of study subjects in KNHANES (2009–2012) by coffee consumption and age group (≥65).

	Coffee consumption					P-value[1)]
	Total (n=2184)	None (n=429)	Monthly (n=122)	Weekly (n=383)	Daily (N=1250)	
**Age group (≥65)**						
Age (year), median [IQR]	71 [68, 75]	72 [68, 76]	72 [68, 75]	71 [68, 75]	71 [68, 75]	0.0173
Male, n (%)	1040 (47.62%)	145 (33.80%)	43 (35.25%)	173 (45.17%)	679 (54.32%)	<.0001
Education, n (%)						<.0001

Less than middle school	1632 (74.73%)	362 (84.38%)	93 (76.23%)	296 (77.28%)	881 (70.48%)	
High school	345 (15.80%)	41 (9.56%)	23 (18.85%)	53 (13.84%)	228 (18.24%)	
College or more	207 (9.48%)	26 (6.06%)	6 (4.92%)	34 (8.88%)	141 (11.28%)	
Father's education, n (%)						0.4676
Less than middle school	661 (30.27%)	127 (29.60%)	31 (25.41%)	106 (27.68%)	397 (31.76%)	
High school	103 (4.72%)	23 (5.36%)	7 (5.74%)	13 (3.39%)	60 (4.80%)	
College or more	60 (2.75%)	12 (2.80%)	4 (3.28%)	7 (1.83%)	37 (2.96%)	
No/non-formal education	1360 (62.27%)	267 (62.24%)	80 (65.57%)	257 (67.10%)	756 (60.48%)	
Mother's education, n (%)						0.4415
Less than middle school	477 (21.84%)	82 (19.11%)	24 (19.67%)	84 (21.93%)	287 (22.96%)	
High school	31 (1.42%)	8 (1.86%)	2 (1.64%)	2 (0.52%)	19 (1.52%)	
College or more	7 (0.32%)	0 (0.00%)	0 (0.00%)	1 (0.26%)	6 (0.48%)	
No/non-formal education	1669 (76.42%)	339 (79.02%)	96 (78.69%)	296 (77.28%)	938 (75.04%)	
Monthly house income[4)], median [IQR]	100 [54, 233]	95 [50, 200]	87 [42, 270]	100 [50, 225]	108 [57, 250]	0.0225
Sleeping duration (hours), n (%)						0.0031
< 6	595 (27.24%)	144 (33.57%)	43 (35.25%)	101 (26.37%)	307 (24.56%)	
6–7	468 (21.43%)	95 (22.14%)	19 (15.57%)	90 (23.50%)	264 (21.12%)	
7–8	513 (23.49%)	89 (20.75%)	23 (18.85%)	96 (25.07%)	305 (24.40%)	
≥8	608 (27.84%)	101 (23.54%)	37 (30.33%)	96 (25.07%)	374 (29.92%)	
High perceived stress, n (%)	470 (21.52%)	109 (25.41%)	29 (23.77%)	96 (25.07%)	236 (18.88%)	0.0063
Exposure to indoor secondhand smoke						
At work, n (%)	42 (1.92%)	7 (1.63%)	7 (5.74%)	5 (1.31%)	23 (1.84%)	0.0409
At home, n (%)	24 (1.10%)	8 (1.86%)	1 (0.82%)	0 (0.00%)	15 (1.20%)	0.039
Current smoking, n (%)	272 (12.45%)	27 (6.29%)	6 (4.92%)	29 (7.57%)	210 (16.80%)	<.0001
Heavy drinking[5)], n (%)	293 (13.42%)	32 (7.46%)	15 (12.30%)	51 (13.32%)	195 (15.60%)	0.0002

Difficulties controlling alcohol use, n (%)	106 (4.85%)	11 (2.56%)	2 (1.64%)	21 (5.48%)	72 (5.76%)	0.013
Having drinking-related problems, n (%)	38 (1.74%)	3 (0.70%)	0 (0.00%)	8 (2.09%)	27 (2.16%)	0.0868
Menopause (females)						<.0001
No	21 (1.84%)	4 (1.41%)	2 (2.53%)	5 (2.38%)	10 (1.75%)	
Yes	1123 (98.16%)	280 (98.59%)	77 (97.47%)	205 (97.62%)	561 (98.25%)	
Hypertension, n (%)	1089 (49.86%)	219 (51.05%)	63 (51.64%)	197 (51.44%)	610 (48.80%)	0.7205
Diabetes mellitus, n (%)	406 (18.59%)	87 (20.28%)	23 (18.85%)	81 (21.15%)	215 (17.20%)	0.2445
Anemia, n (%)	243 (12.14%)	49 (13.42%)	12 (10.91%)	46 (12.96%)	136 (11.60%)	0.7352
Kidney failure, n (%)	14 (0.64%)	3 (0.70%)	0 (0.00%)	4 (1.04%)	7 (0.56%)	0.6653
Thyroid disorder, n (%)	45 (2.06%)	14 (3.26%)	4 (3.28%)	8 (2.09%)	19 (1.52%)	0.0949
Tympanic membrane perforation, n (%)	92 (4.21%)	23 (5.36%)	2 (1.64%)	15 (3.92%)	52 (4.16%)	0.3396
Cholesteatoma, n (%)	58 (2.66%)	15 (3.50%)	5 (4.10%)	6 (1.57%)	32 (2.56%)	0.2376
Otitis media with effusion, n (%)	11 (0.50%)	2 (0.47%)	0 (0.00%)	2 (0.52%)	7 (0.56%)	>.9999

1) P-values from Fisher exact test (binary covariates), chi-square test (>3 categories) and Wilcoxon rank-sum test (continuous covariates); 2) Monthly household income (10,000 Korean Won); 3) Heavy drinking: more than 3 drinks on average and more than twice weekly.

Table S4. Degree of unilateral hearing loss by coffee consumption.

	Frequency of coffee consumption				
	**Total**	**Rarely**	**Monthly**	**Weekly**	**Daily**
**Age group (19-39)**					
More than mild	150 (3.2%)	21 (3.3%)	11 (3.6%)	21 (2.4%)	97 (3.4%)
More than moderate	55 (1.2%)	11 (1.7%)	2 (0.7%)	6 (0.7%)	36 (1.3%)
More than severe	25 (0.5%)	3 (0.5%)	1 (0.3%)	3 (0.3%)	18 (0.6%)
Profound	3 (0.1%)	0 (0.0%)	0 (0.0%)	0 (0.0%)	3 (0.1%)
Total	4633	634	304	873	2822
**Age group (40-64)**					
More than mild	673 (10.1%)	67 (10.2%)	21 (6.8%)	106 (11.8%)	479 (10.0%)
More than moderate	332 (5.0%)	40 (6.1%)	15 (4.9%)	47 (5.2%)	230 (4.8%)
More than severe	94 (1.4%)	22 (3.4%)	8 (2.6%)	7 (0.8%)	57 (1.2%)
Profound	30 (0.5%)	6 (0.9%)	0 (0.0%)	4 (0.4%)	20 (0.4%)
Total	6631	656	308	899	4768
**Age group (≥65)**					
More than mild	320 (14.7%)	70 (16.3%)	24 (19.7%)	59 (15.4%)	167 (13.4%)
More than moderate	311 (14.2%)	71 (16.6%)	18 (14.8%)	58 (15.1%)	164 (13.1%)
More than severe	97 (4.4%)	19 (4.4%)	6 (4.9%)	17 (4.4%)	55 (4.4%)
Profound	37 (1.7%)	8 (1.9%)	1 (0.8%)	4 (1.0%)	24 (1.9%)
Total	2184	429	122	383	1250

Table S5. Degree of bilateral hearing loss by coffee consumption.

	Frequency of coffee consumption				
	**Total**	**Rarely**	**Monthly**	**Weekly**	**Daily**
**Age group (19-39)**					
More than mild	45 (1.0%)	6 (1.0%)	0 (0.0%)	8 (0.9%)	31 (1.1%)
More than moderate	8 (0.2%)	1 (0.2%)	0 (0.0%)	3 (0.3%)	4 (0.1%)
More than severe	1 (0.0%)	0 (0.0%)	0 (0.0%)	0 (0.0%)	1 (0.0%)
Profound	0	-	-	-	-
Total	4633	634	304	873	2822
**Age group (40-64)**					
More than mild	854 (12.9%)	113 (17.2%)	47 (15.3%)	119 (13.2%)	575 (12.1%)
More than moderate	192 (2.9%)	31 (4.7%)	18 (5.8%)	33 (3.7%)	110 (2.3%)
More than severe	26 (0.4%)	3 (0.5%)	2 (0.7%)	7 (0.8%)	14 (0.3%)
Profound	9 (0.1%)	1 (0.2%)	0 (0.0%)	3 (0.3%)	5 (0.1%)
Total	6631	656	308	899	4768
**Age group (≥65)**					
More than mild	1259 (57.6%)	246 (57.3%)	70 (57.4%)	216 (56.4%)	727 (58.2%)
More than moderate	458 (21.0%)	99 (23.1%)	20 (16.4%)	79 (20.6%)	260 (20.8%)
More than severe	58 (2.7%)	16 (3.7%)	3 (2.5%)	13 (3.4%)	26 (2.1%)
Profound	29 (1.3%)	9 (2.1%)	0 (0.0%)	8 (2.1%)	12 (1.0%)
Total	2184	429	122	383	1250

Table S6. Odds ratio and 95% confidence intervals by coffee consumption for degree of unilateral hearing loss.

		Mild		Moderate		Severe		Profound	
		**OR (95% CI)**	**P-value**	**OR (95% CI)**	**P-value**	**OR (95% CI)**	**P-value**	**OR (95% CI)**	**P-value**
**Age 19-39**									
Univariable	Rarely	reference		reference		reference			
	Monthly	1.10 (0.52, 2.30)	0.8090	0.38 (0.08, 1.70)	0.2039	0.69 (0.07, 6.70)	0.7523		
	Weekly	0.72 (0.39, 1.33)	0.2931	0.39 (0.14, 1.07)	0.0664	0.73 (0.15, 3.61)	0.6946	-[1)]	
	Daily	1.04 (0.64, 1.68)	0.8756	0.73 (0.37, 1.45)	0.3688	1.35 (0.40, 4.60)	0.6310		
Multivariable[2)]	Rarely	Reference		reference		reference			
	Monthly	0.92 (0.42, 2.01)	0.8424	0.42 (0.09, 1.99)	0.2768	0.95 (0.19, 4.83)	0.9495		
	Weekly	0.62 (0.33, 1.18)	0.1434	0.45 (0.16, 1.28)	0.1338	0.72 (0.21, 2.49)	0.6058	-	
	Daily	0.92 (0.56, 1.51)	0.7409	0.76 (0.36, 1.59)	0.4595	1.13 (0.42, 3.03)	0.8048		
**Age 40-64**									
Univariable	Rarely	Reference		reference		reference		reference	
	Monthly	0.64 (0.39, 1.07)	0.0900	0.79 (0.43, 1.45)	0.4442	0.77 (0.34, 1.75)	0.5294	0.16 (0.01, 2.90)	0.2164
	Weekly	1.18 (0.85, 1.63)	0.3290	0.85 (0.55, 1.31)	0.4611	0.23 (0.10, 0.53)	0.0007	0.50 (0.15, 1.68)	0.2643
	Daily	0.98 (0.75, 1.29)	0.8938	0.78 (0.55, 1.10)	0.1603	0.35 (0.21, 0.57)	<.0001	0.43 (0.18, 1.05)	0.0636
Multivariable	Rarely	reference		reference		reference		reference	

	Monthly	0.74 (0.44, 1.25)	0.2599	0.80 (0.42, 1.52)	0.4891	0.83 (0.36, 1.93)	0.6667	0.14 (0.01, 1.73)	0.1264
	Weekly	1.32 (0.94, 1.86)	0.1146	0.86 (0.54, 1.36)	0.5141	0.25 (0.10, 0.59)	0.0017	0.52 (0.18, 1.47)	0.2178
	Daily	1.17 (0.87, 1.56)	0.3039	0.85 (0.59, 1.23)	0.3865	0.38 (0.22, 0.64)	0.0003	0.44 (0.20, 0.96)	0.0399
**Age ≥65**									
Univariable	Rarely	reference		reference		reference		reference	
	Monthly	1.26 (0.75, 2.10)	0.3840	0.87 (0.50, 1.53)	0.6344	1.12 (0.44, 2.86)	0.8189	0.44 (0.05, 3.51)	0.4347
	Weekly	0.93 (0.64, 1.36)	0.7226	0.90 (0.62, 1.31)	0.5841	1.00 (0.51, 1.96)	0.9946	0.56 (0.17, 1.86)	0.3401
	Daily	0.79 (0.58, 1.07)	0.1297	0.76 (0.56, 1.03)	0.0778	0.99 (0.58, 1.69)	0.9799	1.03 (0.46, 2.31)	0.9425
Multivariable	Rarely	reference		reference		reference		reference	
	Monthly	1.06 (0.61, 1.86)	0.8259	0.89 (0.48, 1.66)	0.7070	1.20 (0.45, 3.17)	0.7211	0.54 (0.11, 2.55)	0.4337
	Weekly	0.84 (0.56, 1.26)	0.3934	0.97 (0.64, 1.47)	0.8765	0.77 (0.36, 1.62)	0.4850	0.52 (0.17, 1.59)	0.2529
	Daily	0.78 (0.56, 1.08)	0.1269	0.85 (0.60, 1.19)	0.3360	0.88 (0.50, 1.58)	0.6731	0.81 (0.38, 1.74)	0.5919

1) Analysis was not performed because of less than 5 hearing losses of the definition.

2) Adjusted for age, sex, education, parents' education, perceived stress, exposure to indoor secondhand smoke, current smoking, heavy drinking, drinking-related problem, menopause, history of hypertension, diabetes mellitus, anemia, kidney failure, thyroid disorder, tympanic membrane perforation, cholesteatoma and otitis media with effusion.

Table S7. Odds ratio and 95% confidence intervals by coffee consumption for degree of bilateral hearing loss.

		Mild		Moderate		Severe		Profound	
		OR (95% CI)	P-value	OR (95% CI)	P-value	OR (95% CI)	P-value	OR (95% CI)	P-value
**Age 19-39**									
Univariable	Rarely	reference		reference					
	Monthly	0.16 (0.01, 2.84)	0.2111	0.69 (0.03, 17.15)	0.8231				
	Weekly	0.95 (0.34, 2.65)	0.9214	1.70 (0.25, 11.55)	0.5880	-[1)]		-	
	Daily	1.09 (0.47, 2.55)	0.8406	0.67 (0.11, 4.29)	0.6765				
Multivariable[2)]	Rarely	reference		reference					
	Monthly	0.19 (0.02, 2.52)	0.2101	0.59 (0.06, 6.35)	0.6656				
	Weekly	0.97 (0.36, 2.60)	0.9475	1.53 (0.37, 6.32)	0.5607	-		-	
	Daily	0.90 (0.39, 2.11)	0.8153	0.74 (0.19, 2.94)	0.6715				
**Age 40-64**									
Univariable	Rarely	reference		reference		reference		reference	
	Monthly	0.87 (0.60, 1.25)	0.4446	1.25 (0.69, 2.28)	0.4604	1.42 (0.24, 8.56)	0.7002	0.71 (0.03, 17.52)	0.8331
	Weekly	0.73 (0.55, 0.97)	0.0296	0.77 (0.47, 1.27)	0.3023	1.71 (0.44, 6.63)	0.4391	1.71 (0.25, 11.60)	0.5849
	Daily	0.66 (0.53, 0.82)	0.0002	0.48 (0.32, 0.72)	0.0004	0.64 (0.18, 2.24)	0.4855	0.51 (0.08, 3.08)	0.4584
Multivariable[1)]	Rarely	reference		reference		reference		reference	

	Monthly	0.82 (0.55, 1.24)	0.3532	1.19 (0.62, 2.27)	0.6051	1.00 (0.20, 5.09)	0.9999	0.88 (0.09, 8.41)	0.9105
	Weekly	0.72 (0.53, 0.98)	0.0340	0.74 (0.43, 1.26)	0.2624	1.47 (0.50, 4.33)	0.4888	1.82 (0.45, 7.44)	0.4048
	Daily	0.70 (0.55, 0.90)	0.0048	0.51 (0.33, 0.78)	0.0021	0.62 (0.23, 1.67)	0.3478	0.57 (0.15, 2.11)	0.3965
**Age ≥65**									
Univariable	Rarely	reference		reference		reference		reference	
	Monthly	1.00 (0.67, 1.50)	0.9946	0.65 (0.39, 1.11)	0.1155	0.65 (0.19, 2.27)	0.5005	0.18 (0.01, 3.16)	0.2413
	Weekly	0.96 (0.73, 1.27)	0.7849	0.87 (0.62, 1.21)	0.3997	0.91 (0.43, 1.91)	0.7972	1.00 (0.39, 2.56)	0.9967
	Daily	1.03 (0.83, 1.29)	0.7675	0.88 (0.67, 1.14)	0.3212	0.55 (0.29, 1.03)	0.0627	0.45 (0.19, 1.05)	0.0637
Multivariable1)	Rarely	reference		reference		reference		reference	
	Monthly	1.09 (0.70, 1.70)	0.6889	0.72 (0.40, 1.29)	0.2720	0.79 (0.26, 2.37)	0.6684	0.19 (0.02, 1.96)	0.1610
	Weekly	1.06 (0.78, 1.44)	0.6986	0.90 (0.62, 1.31)	0.5953	0.82 (0.40, 1.69)	0.5891	0.92 (0.40, 2.12)	0.8425
	Daily	1.06 (0.83, 1.36)	0.6258	0.84 (0.62, 1.14)	0.2669	0.37 (0.19, 0.69)	0.0020	0.27 (0.12, 0.60)	0.0014

1) Analysis was not performed because of less than 5 hearing losses of the definition.

2) Adjusted for age, sex, education, parents' education, perceived stress, exposure to indoor secondhand smoke, current smoking, heavy drinking, drinking-related problem, menopause, history of hypertension, diabetes mellitus, anemia, kidney failure, thyroid disorder, tympanic membrane perforation, cholesteatoma and otitis media with effusion.
